# Antiosteoporotic Effects and Proteomic Characterization of the Target and Mechanism of an Er-Xian Decoction on Osteoblastic UMR-106 and Osteoclasts Induced From RAW264.7

**DOI:** 10.3390/molecules15074695

**Published:** 2010-07-05

**Authors:** Zheng Zhu, Li-Ming Xue, Ting Han, Lei Jiao, Lu-Ping Qin, Yu-Shan Li, Han-Chen Zheng, Qiao-Yan Zhang

**Affiliations:** 1 School of Pharmaceutical Sciences, China Medical University, No.92 Bei Er Road, He Ping District, Shenyang, 110001, China; 2 School of Pharmacy, Second Military Medical University, 325 Guohe Road, Shanghai, 200433, China; 3 College of Traditional Chinese Material Medica, Shenyang Pharmaceutical University, Number 103 Wen Hua Road, Shen He District, Shenyang, 110016, China

**Keywords:** Er-Xian decoction, osteoporosis, proteomics, osteoblast, osteoclast

## Abstract

Er-Xian Decoction (EXD) has been used for the treatment of osteoporosis disorders, menopausal syndrome, and other aging diseases in the Chinese traditional healthcare system. However, the targets and mechanism of action have not been clarified. This study was designed to investigate the effects and possible target proteins of EXD on osteoblastic UMR-106 cells and osteoclasts induced from RAW264.7 cells using the proteomic analysis technique. We found that EXD at a concentration of 50–200 μg/mL significantly enhanced osteoblastic UMR-106 cell proliferation, alkaline phosphatase (ALP) activity and formation of bone nodules, and decreased tartrate-resistant acid phosphatase (TRAP) activity and the bone resorption action of osteoclasts induced from RAW 264.7 cells. In EXD-treated osteoblasts, there were increases in the expression of heat-shock protein 1, high mobility group protein (Hmgb1), acidic ribosomal phosphoprotein P0, histone 2, carbonyl reductase 1, ATP synthase, aldolase A, and Rho GDP dissociation inhibitor (GDI)-alpha; and reduction in the expression of carbonic anhydrase 3, prohibitin, hemiferrin, far upstream element (FUSE)-binding protein. In EXD-treated osteoclasts, there were increases in the expression of vimentin, protein disulfide isomerase associated 3 and alpha-fetoprotein; and reduction in the expression of calnexin. These results indicated that EXD modulates bone metabolism through regulation of osteoblastic proliferation, apoptosis, and cell activation, and osteoclastic protein folding and aggregation.

## 1. Introduction

Er-Xian Decoction (EXD), a traditional Chinese medicine, has been used for the treatment of osteoporosis disorders[1] menopausal syndrome, and aging diseases in the past 50 years [2]. We previously have reported that EXD has positive effects on bone in ovariectomized rats [3], and the flavonoids, saponins, alkaloids and anthraquinines present in EXD are responsible for its antiosteoporotic activity[4]. These findings helped to clarify the antiosteoporotic activity of EXD and detail its chemical constituents, but as the mechanisms of action of EXD are incompletely understood, this has hindered its application in healthcare systems worldwide.

In bone remodeling, osteoclasts resorb aged or damaged bone, leaving space for osteoblasts to make new bone. The proliferation, differentiation, and apoptosis of osteoclasts and osteoblasts, which determines the amount of bone and its microarchitecture, can be regulated by drugs[5] . In the present study, we found that EXD can stimulate osteoblastic UMR-106 cell proliferation, alkaline phosphatase (ALP) activity and bone formation, and decrease tartrate-resistant acid phosphatase (TRAP) activity and bone resorption of osteoclasts derived from RAW264.7 cells. Proteomic technology was used to seek differential protein expression in osteoblasts and osteoclasts treated with EXD to comprehensively analyze the anti-osteoporotic targets and mechanism.

## 2. Results and Discussion

### 2.1. Stimulatory effects on osteoblastic UMR-106 cells

**Figure 1:**
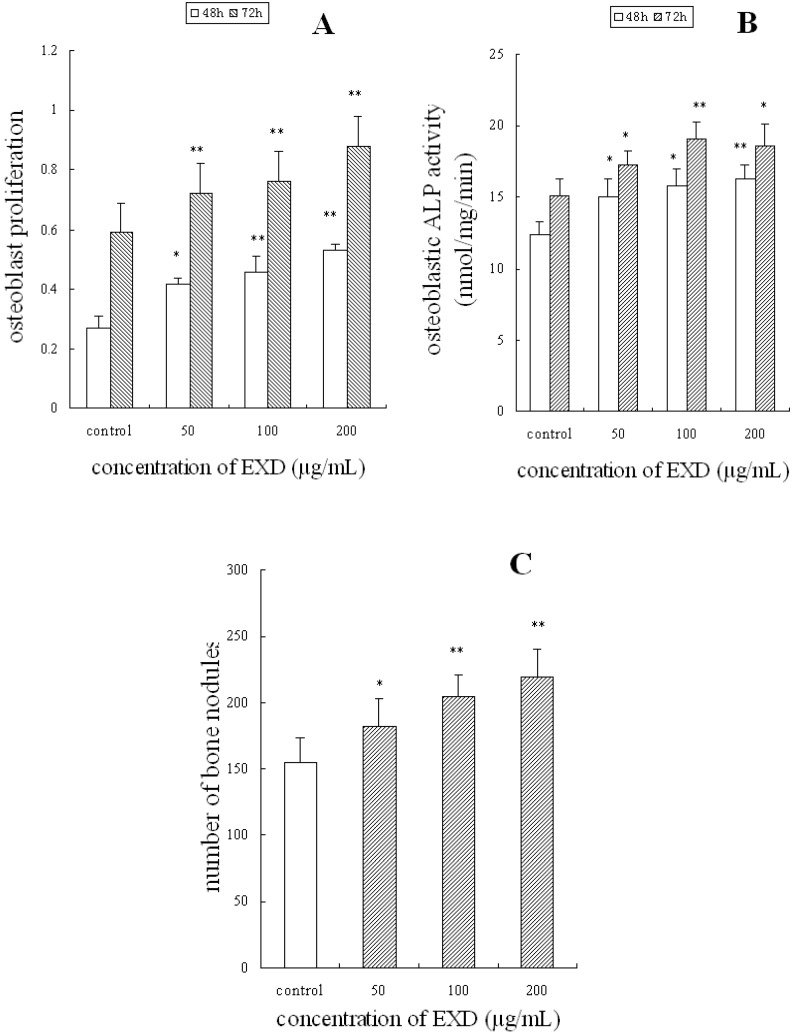
Effects of EXD on osteoblastic UMR-106 cells. Osteoblastic UMR-106 cells were treated with EXD at concentrations of 50, 100, or 200 μg/mL for 48 h and 72 h (proliferation and ALP activity) or 14 days (formation of bone nodules). A: osteoblast proliferation; B: osteoblastic ALP activity; C: formation of bone nodules. *P < 0.05; **P < 0.01 compared with control, *n* = 8, mean±SD.

Osteoblastic bone formation is thought to be mediated by two different processes: one is the formation of new osteoblasts, and the other is the activity of osteoblasts to produce bone matrix. ALP is an important enzyme in bone remodeling, which promotes mineralization of bone matrix. As shown in Figure 1 A B after treat ment with EXD at concentrations of 50, 100, or 200 μg/mL for 48 h and 72 h, the proliferation and ALP activity of osteoblastic UMR-106 cell were signifficantly increased compared with that of control (P < 0.01). Further investigation showed that EXD increased the formation of bone noudles of osteoblastic UMR-106 cells (Figure 1C). This indicated that EXD facilitates bone formation not only through increasing the number of osteoblasts, but also by increasing osteoblastic activity, and the formation and mineralization of bone matrix.

### 2.2. Inhibitory effects on osteoclasts induced from RAW264.7

**Figure 2:**
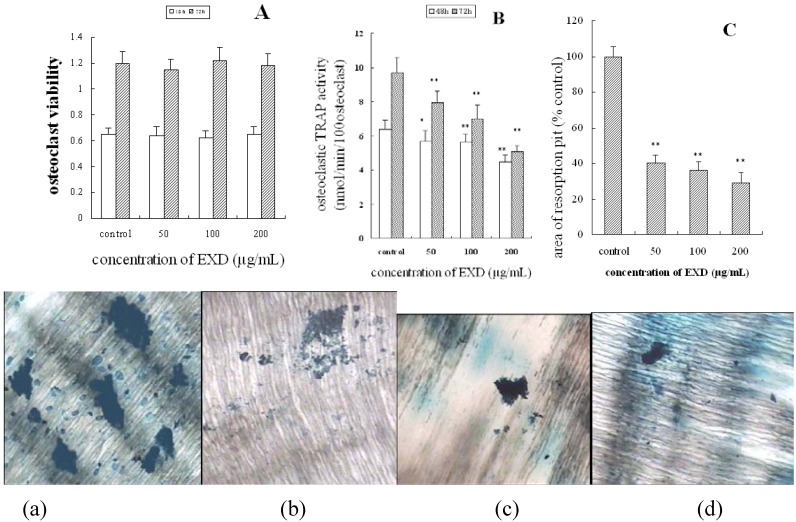
Effects of EXD on osteoclasts induced from RAW264.7 cell. Osteoclasts were treated with EXD at concentration of 50, 100, or 200 μg/mL for 48 h and 72 h (viability and TRAP activity) or 12 days (bone resorption). A: osteoclast viability; B: osteoclastic TRAP activity; C: bone resorption, a, b, c, d respectively are bone resorption pit produced by osteoclast induced from RAW264.7 treated with control or EXD at concentration of 50 μg/mL, 100 μg/mL and 200 μg/mL. *P < 0.05; **P < 0.01 compared with control, *n* = 8, mean ± SD.

The activity of TRAP is directly related to osteoclastic bone resorption. EXD inhibited the TRAP activity of osteoclasts at 50–200 μg/mL, and decreased TRAP by 30.9% and 47.4%, respectively, compared with the control at 100 μg/mL and 200 μg/mL for 72 h (Figure 2B). As shown in Figure 2C the RAW264.7 cells were induced to differentiate into osteoclast by RANKL and M-CSF the mature osteoclast resorbed the bone matrix and formed bone resorption pit on bone dental slices. After osteoclasts were treated with EXD at 50 μg/mL 100 μg/mL and 200 μmol/mL for 12 d, the bone resorption pit area on the surface of bone slices were respectively (40.5 ± 4.2)%, (36.2 ± 4.5)% and (29.1±5.8)% compared with control (Figure 2C). These results indicated that EXD caused a drastic and significant dose-dependent decrease in bone resorption pit at the concentration of 50 μg/mL, 100 μg/mL and 200 μmol/mL. Cytotoxic effects of EXD were then analyzed. Osteoclasts induced from RAW264.7 were treated with increasing amount of EXD extracts (50–200 μg/mL) for 48 and 72 h, and the viability of the cells was examined by the colorimetric MTT assay. As shown in Figure 2A, 50, 100 and 200 μg/mL of EXD extracts did not cause any cytotoxic effect on the total cell population (more than 95% cells were induced into TRAP-positive cells). This indicated that inhibitory effects of EXD on TRAP activity and bone resorption of osteoclast were not caused by decreasing cell viability.

### 2.3. Protein expression profile in EXD-treated osteoblasts and osteoclasts

**Figure 3:**
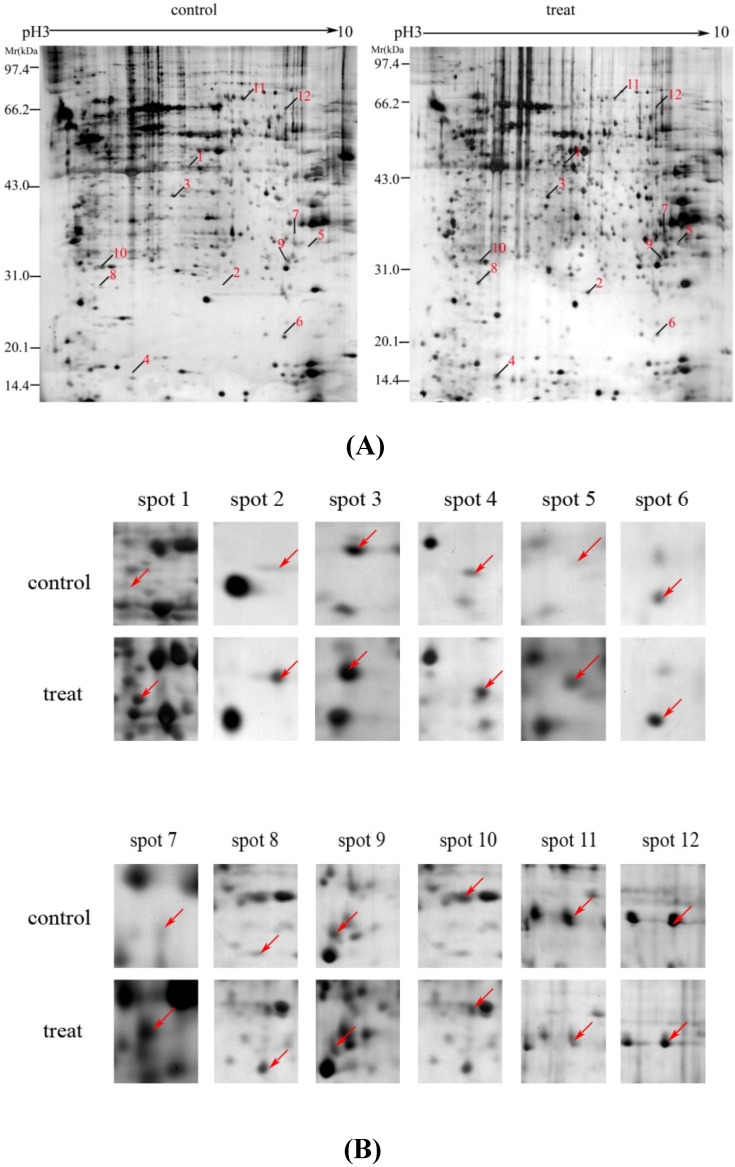
The proteome maps (2-DE images) of osteoblastic UMR 106 cells. Molecular weight (MW, kDa) and isoelectric point (*pI*) are indicated along the y- and x-axis, respectively (A). Selected regions show significant differences in the protein expression profile of osteoblasts (B) between untreated and those treated with 100 µg/mL of EXD for 48 h. Arrows indicate proteins that are differentially expressed absolutely. The gel images are representative gels from among nine replicate gels collected from three independent experiments. Differentially expressed protein spots indicated by lines and numbers were chosen for MALDI-TOF MS/MS analysis, outlined in Table 1.

2-DE and gel sliver staining were conducted to further investigate the differential protein expression between EXD-treated and -untreated osteoblasts. Representative 2-DE gel images for UMR-106 cells are shown in Figure 3A. Gel images were analyzed via PD-Quest software, 1087 protein spots could be resolved from each gel. Twenty-four protein spots were found to be significantly regulated in the EXD-treated group compared with control. Twelve of these 24 spots exhibited a more than two fold increase or decrease in abundance as observed in all replicate gels. These 12 regulated proteins were indicated by the arrowed spots in Figure 3A and by the expanded plots in Figure 3B, and were cut from the gels for further identification by matrix-assisted laser desorption/ionization-time of flight/mass spectrometry (MALDI-TOF/MS) analysis.

**Figure 4:**
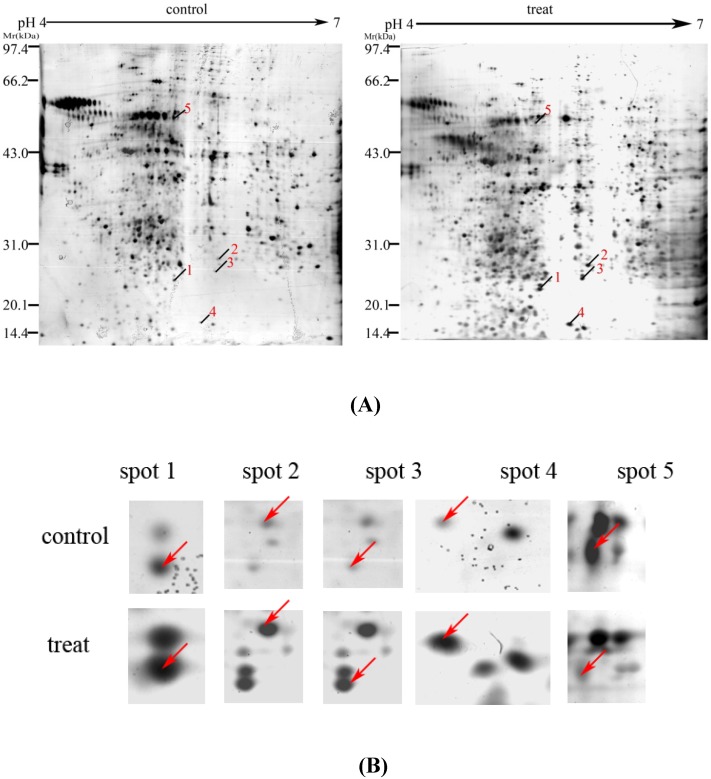
The proteome maps (2-DE images) of osteoclast induced from RAW 264.7 cells. Molecular weight (MW, kDa) and isoelectric point (*pI*) are indicated along the y- and x-axis, respectively (A). Selected regions show significant differences in protein expression profile of osteoclasts (B) between untreated and treated with 100 µg/mL of EXD for 48 h. Arrows indicate proteins that are differentially expressed absolutely. The gel images are the representative gel among nine replicate gels collected from three independent experiments. Differentially expressed protein spots indicated by lines and numbers were chosen for MALDI-TOF MS/MS analysis, outlined in Table 1.

Representative 2-DE gel images for osteoclasts induced from RAW264.7 cells are shown in Figure 4A. Gel images were analyzed via PD-Quest software, and 899 protein spots could be resolved from each gel. Seventeen protein spots were found to be significantly regulated in the EXD-treated group compared with control. Five of these 17 spots exhibited a more than twofold increase or decrease in abundance as observed in all triplicate gels. These five regulated proteins were indicated by the arrowed spots in Figure 4A and by the expanded plots in Figure 4B, and were cut from the gels for further identification by MALDI–TOF /MS analysis. 

**Table 1:** The summary of differentially expressed proteins in osteoblastic UMR 106 cells and osteoclast induced from RAW264.7 cell treated with EXD.

Spot	NCBI Accession number	Target protein	Theoretical Mr (kDa)/pI	Protein Score	Matching peptides (no.)	Fold difference
**Osteoblastic UMR 106 cells**						
1	11560024	Heat shock protein 1	60.9/5.91	431	11	+2.28
2	754208	High mobility group protein(Hmgb1)	24.9/5.62	58	10	+2.64
3	1169376	Acidic ribosomal phosphoprotein P0	34.2/5.91	363	15	+2.18
4	27693390	histone 2	13.9/10.31	114	4	+5.14
5	76779821	Carbonyl reductase 1(NADPH)	30.6/8.22	329	16	+2.02
6	9506411	ATP synthase	18.9/6.17	230	10	+2.17
7	6978487	Aldolase A	39.3/8.31	304	10	+2.43
8	31982030	Rho GDP dissociation inhibitor (GDI) alpha	23.4/5.12	520	9	+2.79
9	31377484	Carbonic anhydrase 3	29.4/6.89	135	9	-2.70
10	6679299	Prohibitin	29.8/ 5.57	766	14	-3.75
11	28849947	Hemiferrin, transferrin-like protein	24.1/7.86	105	6	-2.22
12	83320094	Far upstream element (FUSE) binding protein	67.2/7.18	344	21	-2.50
**Osteoclasts induced from RAW 264.7 cells**						
1	2078001	Vimentin	51.5/4.96	138	7	+3.09
2	112293264	Protein disulfide isomerase associated 3	56.6/5.88	66	7	+6.95
3	112293264	Protein disulfide isomerase associated 3	56.6/5.88	59	5	+4.47
4	191765	Alpha-fetoprotein	47.2/5.47	77	3	+13.03
5	683793	Calnexin	64.9/4.48	86	2	-2.19

**Figure 5:**
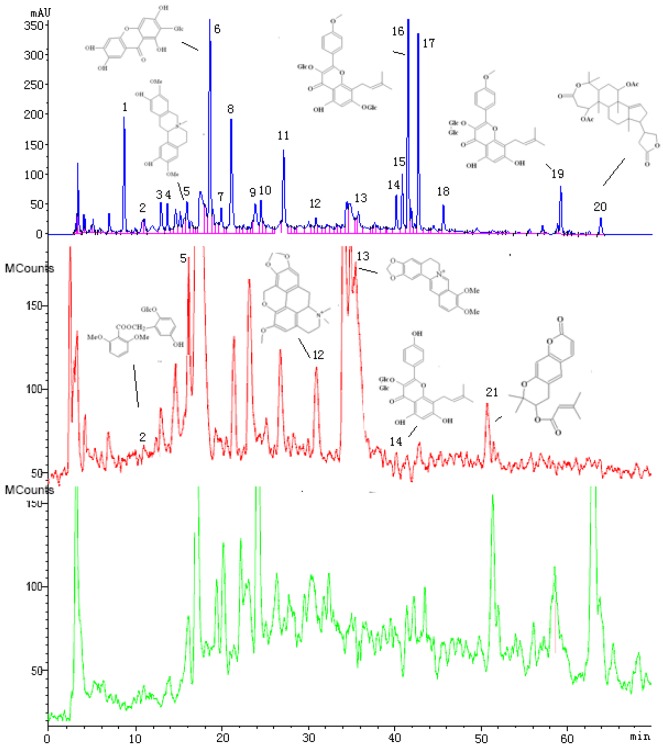
Chromatograms of the EXD extracts with: (A) HPLC (280 nm); (B) MS–TIC (positive ion mode); and (C) MS–TIC (negative ion mode) detection. The peaks in chromatogram were identified by LC–DAD–MS/MS as follows: 2: curculigoside; 5: phellodendrine; 6: mangiferin; 12: thalphenine; 13: berberine; 14: baohuoside; 16: icariin; 19: anhydroicaritin; 20: kihadalactone B; 21: decursin. After comparing the chromatography of EXD with that of individual crud drugs, 1 was the representative peak of *Anemarrhena asphodeloides*; 2 and 11 were the representative peaks of *Curculigo orchioides*; 3, 4, 7, 8, 9 were the representative peaks of *Phellodendron chinense*; 10 was the representative peak of *Angelica sinensis*, and the 12, 14, 15, 16, 17, 18, 19 were the representative peaks of *Epimedium sagittatum.*

The proteins were identified by PMF and MS/MS using the program MASCOT v. 1.9 against the SWISS-PROT database with GPS explorer software. Protein scores were based on combined MS and MS/MS spectra. The spot labels, whose position on the 2-DE gels were displayed in Figure 4 and Figure 5.

### 2.4. Identification of the differentially expressed proteins

Results of the identification of the selected protein spots are summarized in Table 1 for UMR-106 cells and osteoclasts induced from RAW264.7 cells. The molecular weight and isoelectric point (pI) of each protein spot shown in the tables are theoretical values. The protein score, index code and matching peptides of each spot are also shown in Table 1. In UMR-106 cells, protein spots 1–12 were identified as: (1) heat-shock protein 1; (2) high mobility group protein (Hmgb1); (3) acidic ribosomal phosphoprotein P0; (4) histone 2, H2be; (5) carbonyl reductase 1 (NADPH); (6) ATP synthase; (7) aldolase A; (8) Rho GDP dissociation inhibitor (GDI) alpha; (9) carbonic anhydrase 3; (10) prohibitin; (11) hemiferrin, transferrin-like protein; and (12) far upstream element (FUSE) binding protein. For osteoclasts induced from RAW264.7 cells, protein spots 1–5 were identified as: (1) vimentin; (2) protein disulfide isomerase associated 3; (3) protein disulfide isomerase associated 3; (4) alpha-fetoprotein; and (5) calnexin.

EXD contributes significantly to the prevention or treatment of the development of bone loss induced by ovariectomy in rats [3]. Consistent with previous studies, this investigation found that EXD can stimulate osteoblast proliferation, ALP activity and formation of bone nodules, and inhibit osteoclast TRAP activity and bone resorption. The molecular mechanisms of how EXD reduces bone loss are unclear. This study implemented a proteomic scheme to search for the differentially expressed proteins in osteoblasts and osteoclasts affected by EXD. Twelve proteins that may be the target of EXD in osteoblasts, and four proteins that may be the target in osteoclasts were found in the present study.

EXD increased the proliferation of osteoblastic UMR-106 cells. In UMR-106 cells treated with EXD, six proteins associated with the viability and proliferation of cells showed changed expression levels. Heat-shock protein 1 supports cell survival [6]. Hmgb1, which are chromatin proteins, distort, bend or modify the structure of DNA complexes with transcription factors or with histones, is a bone resorption signal within the marrow, and intramembranous and endochondral osteoblasts [7]. Acidic ribosomal phosphoprotein P0 has an important role in the ribosomal “stalk” structure, which is essential for the ribosome to interact with elongation factors [8]. Histone proteins are involved in the transition between active and inactive chromatin states, and its variants influence the replication, transcription, repair and recombination of DNA [9]. Prohibitins regulate transcription, cohesion of sister chromatids, cellular signaling, apoptosis and mitochondrial biogenesis [10]. FUSE-binding protein (FBP) may interact with certain RNA molecules, and participate at various steps in transcription, or in RNA processing, transport or catabolism in the nucleus or cytoplasm. Upon exposure of UMR-106 cells to EXD, the increase of protein expression of heat-shock protein 1, Hmgb1, acidic ribosomal phosphoprotein P0 and histone, and reduction in protein expression of prohibitins and FBP may regulate expression of related regulatory factors, and increase the proliferation and formation of osteoblasts. 

In bone remodeling, osteoblastic bone formation is thought to be mediated by two processes: (i) formation of new osteoblasts; and (ii) the activity of osteoblasts to produce bone matrix (including collagen, ALP, osteocalcin). EXD stimulated the ALP activity of osteoblastic UMR-106 cells, and increased bone noudles formation. Five proteins associated with cell activation and mobility showed changed expression levels. Carbonyl reductases (CBRs) belong to a class of oxidoreductase proteins, and catalyze the NADPH reduction of many biologically and pharmacologically active substrates, including various endogenous and xenobiotic carbonyl compounds [11]. ATP synthase uses the energy contained in a transmembrane proton gradient to drive the synthesis of ATP from ADP and Pi. The extracellular nucleotides (ATP and UTP) respond to mechanical stimulation in different cell systems, including osteoblasts, to prompt osteoblast proliferation, differentiation and matrix formation [12]. Aldolase A protect the cell from oxidative stress, and are involved in the upregulation of ATP production by glycolysis [13]. Rho GDP-dissociation inhibitors (GDI) bind to various Rho proteins and regulate their function [14]. Increase of protein expression of ATP synthase, aldolase A, and GDI-alpha in osteoblasts treated with EXD promotes osteoblast differentiation and bone matrix formation. Carbonic anhydrase catalyzes the production of protons from CO_2_ and H_2_O, which is necessary for bone resorption, and plays a part in bone demineralization. Several groups reported that carbonic anhydrase acts as a mediator of hormones to stimulate bone resorption and osteoclast formation [15]. Reduction of protein expression of carbonic anhydrases in osteoblasts treated with EXD therefore decreased osteoclastic bone resorption in the bone microenvironment. 

In osteoclasts induced from RAW264.7 cells, three proteins are upregulated and one is downregulated by EXD. Vimentin, which is a filament protein and a mesenchymal marker protein, helps support the cellular membrane and keeps the nucleus and other organelles in a defined place within the cell [16]. EXD treatment dramatically increased vimentin expression in osteoclasts. There is cytoskeletal remodeling in the process of induction from the RAW 264.7 cells to the osteoclasts. Increase of these cytoskeleton-related proteins may have been the result of differentiation of the RAW 264.7 cell stimulated by RANKL and M-CSF, and these proteins may be direct targets of EXD. Alpha-fetoprotein is a major non-collagenous component of mineralized bone in mammals, favors fibril mineralization by selectively inhibiting the growth of crystals outside the fibril, and regulates osteogenesis in remodeling bone [17]. EXD treatment increased the expression of alpha-fetoprotein by 13.03-fold. That is, in the bone microenvironment, EXD stimulated osteoclasts to produce alpha-fetoprotein and enhance fibril mineralization and bone matrix formation.

Protein disulfide isomerase (PDI) and calnexin, which are abundantly located in the endoplasmic reticulum, catalyze the formation of disulfide bonds of newly synthesized protein, facilitate protein aggregation under certain conditions [18]. EXD increased the expression of PDI by 4.47-fold. This excess expression of PDI does not usually catalyze the formation of disulfide bonds, but promotes protein aggregation. These incorrectly folded and disassembled proteins lead to the increase of apoptosis of osteoclasts. Calnexin represents a “biologic link” between endoplasmic reticulum intralumenal folding events and cytosolic apoptotic signals, corrects protein misfolding, and allows molecules to move to the plasma membrane [19]. EXD decreased the expression of calnexin by 2.19-fold; this decrease may inhibit the folding of proteins and transportation to the plasma membrane, leading to a reduction in enzyme activity related to bone resorption. 

In summary, EXD treatment altered the expression levels of 12 proteins in osteoblastic UMR-106 cells and four proteins in osteoclasts induced from RAW 264.7 cells. Altered protein expression in osteoblasts treated with EXD was involved in cell proliferation and apoptosis, and in the regulatory mechanism of cell activation. The altered proteins in osteoclasts treated with EXD are mainly related with the folding and aggregation of proteins and cytoskeleton formation. These findings somehow clarify the anti-osteoporotic targets and mechanism of action of EXD. Further study is necessary to investigate the effects of major active constituents in EXD on protein expression on osteoblast and osteoclast so as to understand the interaction and synergistic mechanism, explain the viewpoint that TCM produces therapeutic effects at multiple targets through multiple components.

## 3. Experimental

### 3.1. Reagents

Dulbecco’s modified Eagle’s medium (DMEM) and fetal bovine serum (FBS) were purchased from Gibco (Gaithersburg, USA). Methylthiazoletetrazolium (MTT) and dimethyl sulfoxide (DMSO) were purchased from Amersco (USA). Dithiothreitol DTT, urea, indoacetamide, 3-([3-cholamidopropyl] dimethylammonio)-2-hydroxyl-1-propanesulfonate (CHAPS), Bio-Lyte 3/10 Ampholyte, mineral oil, ammonium persulfate and tetramethylethylenediamine (TEMED) were purchased from Bio-Rad (Hercules, CA, USA). Potassium sodium tartrate tetrahydrate, disodium 4-nitrophenylphosphate, Triton X-100, 4-nitrophenol were of domestic AR grade. 

### 3.2. Preparation of EXD and quality analysis

EXD consists of six plant materials, including *Epimedium sagittatum* (Berberidaceae, whole herb), *Curculigo orchioides* (Amaryllidaceae, rhizome), *Anemarrhena asphodeloides* (Liliaceae, rhizome), *Phellodendron chinense* (Rutaceae, bark), *Morinda officinalis* (Rubiaceae, root) and *Angelica sinensis* (Apiaceae, root) in a compositional ratio of 12:12:9:9:10:10. The above six medicinal materials were obtained from Hua Yu Pharmaceutical Company and identified by Professor H.C. Zheng of the Department of Pharmacognosy, School of Pharmacy of the Second Military Medical University, Shanghai, China. Voucher specimens (numbered as 2007091901; 2007091902; 2007091903; 2007091904; 2007091905 and 2007091906, respectively) are available at the herbarium of this department. 

The six medicinal materials in the mixture (250 g) were powdered and extracted with distilled water [3000 mL, 12: 1 (v/w)] at 100 ºC for 2 h. The extraction was repeated twice. The filtrates were concentrated to 1.5 L under reduced pressure, and lyophilized in a freeze drier, and kept at 4 ºC for quality control and molecular studies. The yield of dried extract from the starting crude materials was 17.8%.

The EXD extracts were dissolved in distilled water, adjusted to 200 µg/mL, and subjected to HPLC-DAD-MS/MS analysis in which major peaks were identified as the marker compounds to their originating individual herbs. The HPLC analysis were carried out on an Agilent 1200 series LC instrument (Agilent Technoligies, Palo Alto, CA, USA) including a quaternary pump, vacuum degasser, thermostatic column compartment and a diode array detector (DAD). An Agilent Extend C-18 analytical column (5 μm, 4.6 × 250 mm) was used. The mobile phase consisted of acetonitrile (A) and 0.1% aqueous acetic acid (v/v, B) using a gradient program of 5–50% A in 0–70 min. The flow rate was 0.7 mL/min and column temperature was maintained at 30 ºC. In the proces of MS/MS analysis, data were acquired and processed by Varian 1200 L workstation software. The electrospray capillary potential was set to 35 V. The API housing and drying gas temperatures were kept at 50 ºC and 380 ºC, respectively. Full scan data acquisition was performed from m/z 200 to 800 in both positive and negative MS scan mode. The fingerprint of HPLC and MS of the extract is shown in Figure 1, and ten compounds were identified as curculigoside, phellodendrine, mangiferin, thalphenine, berberine, baohuoside, icariin, anhydroicaritin, kihadalactone B, decursin. The contents of four standard chemicals (icariin, curculigoside, mangiferin and berberine) in extracts (200 µg crude drug /mL) were determined, and their contents were 3.20 µg/mL, 0.83 µg/mL, 2.54 µg/mL and 1.51 µg/mL, respectively.

### 3.3. Proliferation assay, ALP activity and bone nodules of osteoblastic UMR-106 cells

The osteoblastic UMR-106 were suspended in DMEM medium at a density of 3 × 10^4^/mL, an aliquot of cell suspension (100 µL) was added to 96-well culture dishes to culture for 24 h, and then treated with or without EXD (final concentrations 50 µg/mL, 100 µg/mL and 200 µg/mL) for 48 h and 72 h. Then MTT (20 µL, 5 mg/mL) was added and incubated for another 4 h, the medium was then discarded and DMSO (100 µL) was added. Ultraviolet absorbance was measured spectrophotometrically at 540 nm using an ELx-800 universal microplate reader (Bio-Tek) as an indicator of cell proliferation, the within-run and between-run coefficient of variation (CVs) of assay were respectively no more than 1.23% and 2.63%. 

An aliquot of cell suspension of UMR-106 osteoblastic cells (100 µL) was plated at the concentration of 3 × 10^4^ cells/mL in 96-well dishes, and treated with or without EXD (final concentrations 50 µg/mL, 100 µg/mL and 200 µg/mL) for 48 and 72 h. The ALP activity was measured according to the literature [20]. After rinsing the cells twice with cold PBS, 50 mmol/L diolamine (100 µL) and 2.5 mmol/L disodium 4-nitrophenylphosphate (50 µL) were added, and then incubated for 30 min at 37 ºC. To stop the reaction, 0.3 mol/L NaOH (100 µL) was added to each well. The UV absorbance of the samples and standards was measured at 405 nm. Total protein was assayed by the method of Bradford [21]. The activity of alkaline phosphatase was expressed as as nanomoles of *p*-nitrophenol liberated per minute per milligram protein, the within-run and between-run coefficient of variation (CVs) of assay were no more than 2.26% and 3.52%, respectively.

UMR-106 cells were suspended in DMEM medium at the density of 10^5^/mL, an aliquot of cell suspension (500 µL) was added in 24 well culture dish in the presence of 0.1% BSA, 10 nM dexamethasone, 10 mM Sodium β-glycerophosphate and ascorbic acid (50 μg/mL), and treated with EXD for 14 days. Von Kossa staining was carried out to characterize the biological mineralization of differentiated osteoblasts [22]. The osteoblastic cells in culture dish were then fixed with 10% phosphate-buffered formalin (200 µL) for 10 min, and stained with 2% silver nitrate solution (200 µL). Then the culture dish was exposed to direct sunlight for 20 min, rinsed with water. It was then neutralized with sodium thiosulfate (5%) for 3 min, rinsed with water and counterstained with acid fuchsin for 5 min. The plates were washed with deionized water, then twice with 95% ethyl alcohol and 100% ethyl alcohol and finally dried in air. Bone nodules were observed, and numbered in 20 random fields of vision under a microscope, the within-run and between-run coefficient of variation (CVs) of assay were no more than 3.02% and 4.32%, respectively

### 3.4. Induction of osteoclast from RAW264.7 cell

RAW 264.7 cells (ATCC, USA), were incubated in the presence of the receptor activator of NF-κB ligand (RANK-L, 25 ng/mL) and macrophage-colony stimulating factor (M-CSF, 30 ng/mL) in a 96-well plate (5 × 10^3 ^per well) for 72 h. [23,24]. TRAP-positive multinucleated (>3 nuclei) osteoclasts were formed and identified by staining for TRAP (Acid-Phosphatase Kit; Sigma-Aldrich), and more than 95% cells were differentiated into TRAP-positive osteoclasts [25]. 

### 3.5. Assay of the TRAP activity, cytotoxicity and bone resorption of osteoclasts

RAW264.7 cells were suspended in DMEM medium at density of 1 × 10^5^/mL in the presence of RANKL (25 ng/mL) and MCSF (30 ng/mL). An aliquot of cell suspension (100 μL) weas plated in a 96-well culture dish and cultured for 72 h. Then the cells were treated with or without EXD at 50 µg/mL, 100 µg/mL and 200 µg/mL for 48 h and 72 h. The MTT assay was used to evaluate the cytotoxicity of EXD on osteoclasts; the within-run and between-run coefficient of variation (CVs) of assay were no more than 0.67% and 2.79%, Respectively. The activity of TRAP were determined as previously described [26,27]. In brief, after the cells were washed twice with PBS buffer, 0.1% Triton X-100 (20 µL) was added to lyse the cells at room temperature for 15 min, then substrate solution (100 µL, 0.4 g disodium 4-nitrophenylphosphate and 2.0 g potassium tartrate, dissolved in 200 mL of deionized water, adjusting pH to 3.5 with 1 mol/L HCl) was added and incubated at 37 ºC for 30 min. To stop the reaction, 1 mol/L NaOH (100 µL) was added to each well. The UV absorbance was measured at 405 nm. At the same time, the positive cells for TRAP were counted. The activity of TRAP was expressed as nanomoles *p*-nitrophenol per minute per 100 osteoclasts, the within-run and between-run coefficient of variation (CVs) of assay were no more than 1.68% and 3.28%, respectively.

RAW264.7 cells were suspended in DMEM medium at density of 1 × 10^5^/mL in the presence of RANKL (25 ng/mL) and MCSF (30 ng/mL). Cell suspension (100 μL) was seeded into the wells of 96-well culture plates with each well containing a sterilized dental slice, and cultured for 24 h. The cells were treated with or without EXD of 50, 100, 200 μg/mL for 12 days. At the end of incubation, dental slices were fixed in 2.5% glutaraldehyde, ultrasonicated in 0.25 mol/L NH_4_OH to remove adherent cells, and then stained with 1% toluidine blue solution for 10 min at room temperature. After that, the bone slices were sealed in neutral gum. Resorption pits on bone slice were observed and numbered in 20 random fields of vision under a microscope. The images of pit area were collected and quantified with image analysis software (Leica Q550IW, Germany), the within-run and between-run coefficient of variation (CVs) of assay were no more than 3.26% and 2.96%, Respectively.

### 3.6. Extraction of cell protein

UMR-106 cells were incubated at 1 × 10^5^ cells/mL in 6-well dishes and treated with EXD at the final concentration of 100 µg/mL for 48 h. Before the end of culture, cells were rinsed with ice-cold deionized water, treated with lysis buffer (8 M urea, 4% w/v CHAPS, 65 mM DTT, 0.5% w/v Bio-Lyte 3-10 Ampholyte, 1 mM phenylmethylsulfonyl fluoride and a trace of bromophenol blue) in an ice bath for 15 min, and scraped to a tube. Cell lysate in the tube were snap-frozen, subsequently thawed thrice in liquid nitrogen, and centrifuged for 30 min at 14,000 × g at 4 ºC. Supernatant aliquots were stored at -80 ºC. Total protein concentration was determined using the Bradford method [21]. Osteoclasts induced from RAW264.7 cells were incubated at 1 × 10^5^/mL cells in 6-well dishes, and treated with EXD at 100 mg/mL for 48 h. Total cell protein was prepared as above mentioned (BioRad Dimensona gel electrophoresis experiment manual).

### 3.7. Two-dimensional electrophoresis (2-DE) and gel analysis

Two-dimensional electrophoresis was carried out according to the protocols provided by Bio-Rad. Briefly, total protein of each sample (250 µg) was diluted to a final volume of 350 µL in rehydration solution (8 M urea, 4% w/v CHAPS, 65 mM DTT, 0.5% w/v carrier ampholytes and a trace of bromophenol blue). First-dimension isoelectric focusing (IEF) was done using 17 cm, pH 3–10 nonlinear strips (Bio-Rad) for osteoblast experiments, and 17 cm, pH 4–7 linear strips (Bio-Rad) for osteoclast experiments in an immobilized pH gradient isoelectric focusing (IPG IEF) apparatus (Bio-Rad). Active rehydration was carried out at 50 V for 14 h. IEF was gradually carried out from 250 V to 10,000 V and kept constant until focusing for 60,000 Vh. IPG strips were incubated with 50 mM Tris-HCl, pH 8.8, 6 M urea, 30% glycerol, 2% sodium dodecyl sulfate (SDS), 0.002% bromophenol blue, and 2% DTT for 15 min. They were acetylated with 2.5% iodoacetamide for 15 min, and placed on 12% sodium dodecyl sulfate polyacrylamide gel electrophoresis (SDS-PAGE) gels (230 × 200 × 1.5 mm; Bio-Rad vertical system; Bio-Rad) for the second dimension. The parameters were a constant current of 10 mA/gel for 45 min and 25 mA/gel until the front of the bromophenol blue dye reached the bottom of the gel. The 2D-PAGE gels were visualized by silver staining (Bio-Rad). Gels were stored in fixative solution, scanned with Image Scanner (UMAX PowerLook 2100XL) and analyzed with PDQuest7.4 software (Bio-Rad). Paired (control and EXD-treated) protein samples from three independent experiments were analyzed by 2-DE. For each pair of protein samples, triplicate electrophoreses were carried out to ensure reproducibility. Comparisons were made between gel images of protein profiles obtained from the EXD-treated and control groups. 

### 3.8. Matrix-assisted laser desorption/ionization-time of flight/mass spectrometry (MALDI-TOF/MS) analysis and database search

The altered protein spots were excised from the gels and placed into a 96-well microtiter plate. Gel pieces were detained with a solution of 15 mM potassium ferricyanide and 50 mM sodium thiosulfate (1:1) for 20 min at room temperature, washed twice with deionized water, and shrunk by dehydration in acetonitrile. Samples were then swollen in digestion buffer containing 20 mM ammonium bicarbonate and 12.5 ng/μL sequence-grade trypsin (Promega, Madison, WI, USA) at 4 ºC and incubated for 30 min. Gels were digested at 37 ºC for 12 h. Peptides were extracted twice using 0.1% trifluoroacetic acid (TFA) in 50% acetonitrile. After drying, peptide mixtures were redissolved in 50% acetonitrile (0.8 µL), 0.1% TFA containing α-cyano-4-hydroxycinnamic acid (CHCA, 5 mg/mL, Sigma). Each sample was suspended in matrix solution [0.7 mL, CHCA in acetonitrile/water (1:1, v/v) acidified with 0.1% (v/v) TFA]. The solution was spotted on the MALDI stainless-steel target and dried to crystallize. The analysis was done on a 4700 Proteomics Analyzer (TOF/TOFTM; Applied Biosystems, USA) with a 355 nm Nd:YAG laser. The accelerated voltage was operated at 20 kV, and mass resolution was maximized at 1500 Da. The mass range was scanned from 700 Da to 3,200 Da. Myoglobin digested by trypsin was used to calibrate the mass instrument under internal calibration mode. Spectra of the real samples were acquired using a default mode. Protein identification was determined by peptide mass fingerprinting (PMF) and tandem mass spectrometry (MS/MS) via MASCOT (Matrix Science, London, UK) against SWISS_PROT and NCBInr database with GPS explorer software (Applied Biosystems) [28].

### 3.9. Statistical analyses

The experiments were repeated three times in eight replicate samples, and the within-run coefficient of variation (CVs) and between-run CVs were calculated to evaluate the assay method. Data were expressed as mean ± standard deviation. One-way ANOVA followed by Dunnett’s t-test was used for statistical analysis (SPSS 13.0 software, SPSS, USA), and the level of significance was set at *P* < 0.05 (*), *P* < 0.01 (**). 

## 4. Concolusions

In summary, EXD treatment altered the expression levels of 12 proteins in osteoblastic UMR-106 cells and four proteins in osteoclasts induced from RAW 264.7 cells. Altered protein expression in osteoblasts treated with EXD was involved in cell proliferation and apoptosis, and in the regulatory mechanism of cell activation. The altered proteins in osteoclasts treated with EXD are mainly related with the folding and aggregation of proteins and cytoskeleton formation. These findings somehow clarify the anti-osteoporotic targets and mechanism of action of EXD. Further study is necessary to investigate the effects of major active constituents in EXD on protein expression on osteoblast and osteoclast so as to understand the interaction and synergistic mechanism, explain the viewpoint that TCM produces therapeutic effects at multiple targets through multiple components.
